# Long-term survival after multidisciplinary therapy for residual gallbladder cancer with peritoneal dissemination: a case report

**DOI:** 10.1186/s40792-017-0351-x

**Published:** 2017-06-14

**Authors:** Daisuke Kuga, Tomoki Ebata, Yukihiro Yokoyama, Tsuyoshi Igami, Gen Sugawara, Takashi Mizuno, Junpei Yamaguchi, Masato Nagino

**Affiliations:** Division of Surgical Oncology, Department of Surgery, Nagoya University Graduate School of Medicine, 65 Tsurumai-cho, Showa-ku, Nagoya, 466-8550 Japan

**Keywords:** Gallbladder carcinoma, Peritoneal dissemination, Conversion surgery, Chemotherapy, Long-term survival

## Abstract

**Background:**

Although surgical resection is the only curative treatment for gallbladder cancer (GBC), concomitant peritoneal dissemination is considered far beyond the scope of resection. We report a long-term survivor with a residual GBC with multiple peritoneal disseminations who underwent an extended resection after effective chemotherapy.

**Case presentation:**

A 59-year-old male underwent an open cholecystectomy for Mirizzi syndrome at a local hospital. Because of severe inflammation, the gallbladder was perforated during surgery, ending in a piecemeal resection. A pathological examination revealed GBC with positive margins, and the patient was referred to our hospital 1 month after surgery for further treatment. A multidetector-row computed tomography (MDCT) showed three hypoattenuated tumours: a tumour (3.9 cm) at the left medial segment corresponding to the gallbladder bed, a tumour (1.8 cm) around the hepatic flexure of the transverse colon, and a tumour (1.0 cm) at the stump of the cystic duct. Percutaneous needle biopsy was performed, which provided histologic evidence of adenocarcinoma. Thus, the patient had a rapidly progressive local relapse with limited peritoneal dissemination, labelled ycT3N0M1, stage IVB disease according to the UICC system. After the administration of 3 cycles of gemcitabine plus cisplatin combination chemotherapy, the size of all tumours and the CA19-9 level decreased significantly. Since the patient’s general condition and liver function reserve were satisfactory, we decided the initial unresectable scenario to perform surgical therapy. After portal vein embolization, right hepatectomy, resection of the extrahepatic bile duct, partial duodenectomy, and partial colectomy were performed. Operative time was 555 min, and intraoperative blood loss was 1654 mL. Pathologic diagnosis of residual gallbladder carcinoma with peritoneal dissemination was confirmed, and the surgical margins were tumour-free. The patient was discharged on postoperative day 29, with a Clavien-Dindo IIIa complication (abdominal wall abscess). Postoperative adjuvant chemotherapy with tegafur/gimeracil/oteracil was administered during 1 year after surgery. The patient is doing well 6 years after the second surgery without evidence of disease.

**Conclusions:**

Although specific clinical factors were associated with a favourable outcome in this patient, the present report suggests that multidisciplinary therapy may be a promising option in selected patients with distant metastatic GBC.

## Background

Advanced gallbladder cancer (GBC) often exhibits distant metastasis at the time of initial presentation. Among the various modes of distant metastasis, peritoneal dissemination is an extremely devastating disease, with a median survival time (MST) of only 4.8 months [1]. Therefore, patients with this disease are considered as contraindicated for definitive surgery and undergo systemic chemotherapy. Valle et al. [2] reported the results of the ABC-02 trial where the MST of patients with unresectable/recurrent biliary tract cancers who received gemcitabine plus cisplatin (GC) therapy was 11.7 months; no patients survived for more than 3 years. The BT-22 trail, reported by Okusaka et al. [3], demonstrated an identical outcome to GC chemotherapy. Thus, the effect of the first-line treatment with GC remains limited, and long-term survival in patients with disseminated GBC cannot be expected with chemotherapy alone.

Accidental GBC is histologically found after cholecystectomy for either acute or chronic cholecystitis, with a reported incidence of 0.2 to 2.1% [4–8]. In this setting, severe inflammation commonly triggers perforation of the gallbladder, namely, abdominal contamination of the bile that potentially contains floating cancer cells; consequently, seeding metastasis often develops. Treatment strategies in this setting have yet to be standardized and may be a little different from those used in primary disseminated disease.

To our knowledge, few studies of GBCs with peritoneal dissemination have been reported [9]. Here, we report a rare 5-year survivor of a residual GBC with peritoneal dissemination after cholecystectomy.

## Case presentation

A 59-year-old male had undergone an open cholecystectomy after the clinical diagnosis of Mirizzi syndrome at a local hospital. Because of severe inflammation, the gallbladder was perforated during surgery, ending in a piecemeal resection. Pathologically, the fractioned specimen involved moderately differentiated tubular adenocarcinoma invading the subserosal layer with positive margin of the dissection plane. Lymph nodes and cystic duct stump were not sampled. The patient was referred to our hospital 1 month after the cholecystectomy for further treatment.

**Fig. 1:**
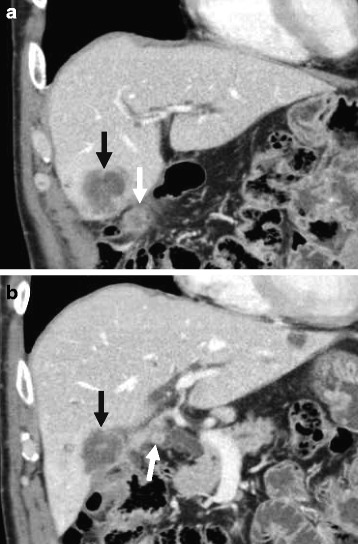
Multidetector-row computed tomography (coronal section images). **a** A tumour with an ill-defined border (*black arrow*) was found in the medial segment (segment 4) 1 month after the initial cholecystectomy. There was a marginal contrast effect in the mass, so the possibility of a postoperative abscess or a carcinoma that had invaded the gallbladder was considered. A mass was also found on the ventral side of the transverse colon, and there was a possibility of peritoneal dissemination (*white arrow*). **b** In addition, another small mass was found at the stump of the cystic duct (*white arrow*)

**Fig. 2:**
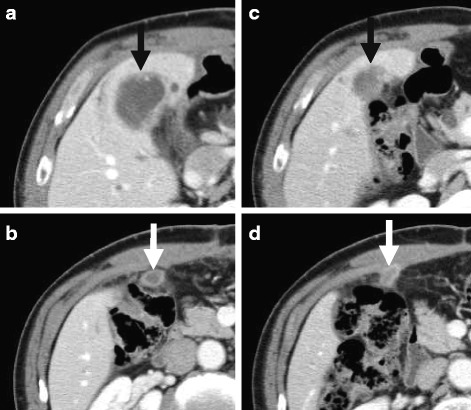
Comparison of the CT images before and after the chemotherapy. The sum of the longest diameter of the tumours decreased from 57 to 36 mm after chemotherapy. Response Evaluation Criteria in Solid Tumours (RECIST) showed a partial response of the effect (**a**, **b** before chemotherapy; **c**, **d** after chemotherapy). *Black arrow* primary tumour, *white arrow* peritoneal dissemination

The patient had undergone a distal gastrectomy with a Billroth II reconstruction for a gastric ulcer at the age of 20 years. At referral, the patient presented as asymptomatic. Liver function tests showed slight abnormalities: total bilirubin, 0.6 mg/dL; aspartate aminotransferase, 15 IU/L; alanine aminotransferase, 15 IU/L; γ-glutamyl transpeptidase, 59 IU/L; and alkaline phosphatase, 236 IU/L. The serum levels of carcinoembryonic antigen (CEA) and carbohydrate antigen 19-9 (CA19-9) were 1.6 ng/mL (normal range, 0–5.0 ng/mL) and 640 IU/L (normal range, 0–37 IU/mL), respectively. The plasma clearance rate of indocyanine green was 0.224. Multidetector-row computed tomography (MDCT) showed an ill-defined tumour, 3.9 cm in diameter, in the left medial segment of the liver (S4, Fig. 1a). The progressively enhancing nature supported the diagnosis of disease relapse, not postoperative abscess. In addition, the second tumour, 1.8 cm in diameter, was found around the hepatic flexure of the transverse colon, which was highly suspicious for peritoneal dissemination (Fig. 1a); the third small mass, 1.0 cm in diameter, was found at the stump of the cystic duct (Fig. 1b). Positron emission tomography-computed tomography (PET-CT) showed a high standardized uptake value (SUV) of the first and second tumours; the maximal values were 6.52 and 7.45, respectively. Biopsy samples taken from the two tumours with a percutaneous approach showed tubular adenocarcinoma. The definitive diagnosis of residual GBC with peritoneal dissemination was reached, classified as ycT3N0M1, stage IVB disease according to the Union for International Cancer Control (UICC) system [10]; the tumours had a rapidly progressive nature after the initial cholecystectomy. Although the tumours seemed to be technically removable with an extended surgical approach, the presence of peritoneal seeding precluded up-front surgery. Therefore, gemcitabine (1000 mg/m^2^) plus cisplatin (25 mg/m^2^) combination therapy (GC) was given on days 1 and 8, every 3 weeks. After 3 cycles of GC therapy, tumour shrinkage was observed and no new lesion emerged. The total sum of the longest diameter of the tumour decreased from 57 to 36 mm (Fig. 2), indicating the effect of a partial response (PR) according to the RECIST (Response Evaluation Criteria in Solid Tumours) system. Additionally, the CA19-9 level decreased from 640 to 124 IU/L. Consequently, resection was scheduled with curative intent.

**Fig. 3:**
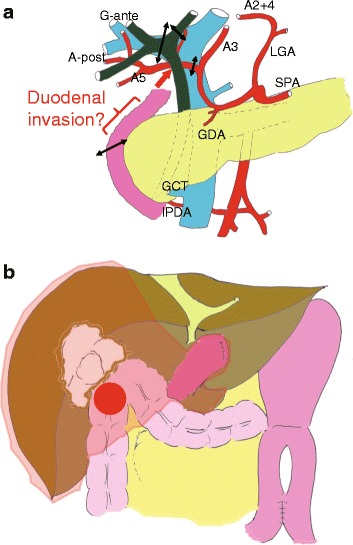
Preoperative schema. **a** The mass involves the duodenum, transverse colon, and common bile duct (*red arrow*). *LGA* left gastric artery, *SPA* splenic artery, *GDA* gastroduodenal artery, *GCT* gastrocolic trunk, *IPDA* inferior pancreaticoduodenal artery. **b** The extent of the resection was estimated in the range of the *red line*. A peritoneal nodule was present at the site indicated by the *red circle*

**Fig. 4:**
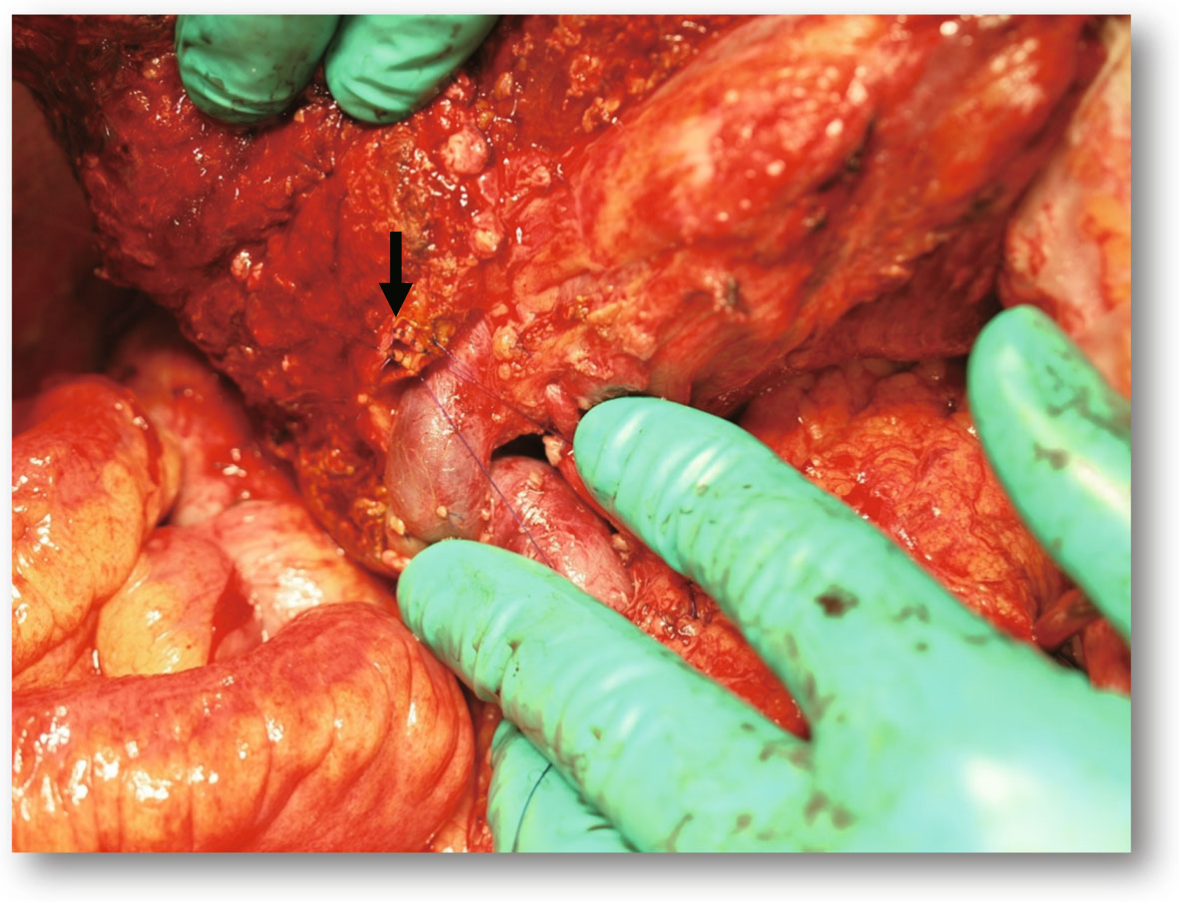
Completion photograph after resection. Extended right hepatectomy, extrahepatic bile duct resection, and partial resection of the duodenum and colon were performed. *Arrow* indicates the stump of the intrahepatic segmental bile ducts

**Fig. 5:**
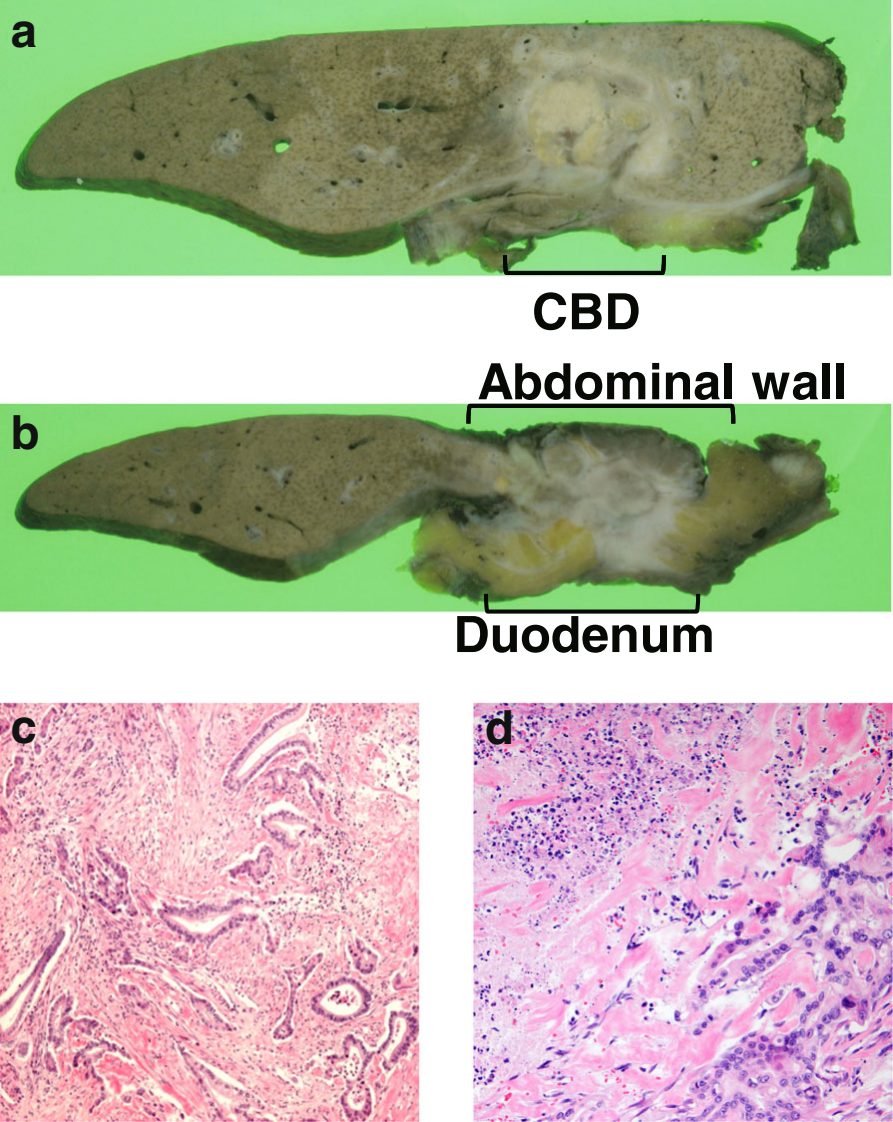
Pathological findings. **a**, **b** Macroscopically, the mass invaded the common bile duct, duodenum, transverse colon, and abdominal wall. **c** Histological examination revealed moderately differentiated adenocarcinoma (residual adenocarcinoma). **d** Histologically, necrosis or degeneration tissue (*top left* corner of the figure) was found in one half to two thirds of the whole area, indicating a grade 1b efficacy

One month after embolization of the right portal vein, a laparotomy was performed. The right upper abdominal quadrant had an extensive adhesion, but ascites and unexpected seeding deposits were not found. As anticipated, one disseminated tumour was found that had invaded the duodenum and transverse colon (Fig. 3). We performed an extended right hepatectomy, extrahepatic bile duct resection, a partial duodenectomy, and a partial transverse colectomy (Fig. 4). Operative time was 555 min, and blood loss was 1654 mL. Histologically, the main tumour was a moderately differentiated adenocarcinoma directly invading the liver with peritoneal dissemination, and the surgical margins were tumour-free (ypT3N0M1, stage IVB, R0 resection). The assessment of the therapeutic effect of chemotherapy was grade 1b (necrosis rate 50–66%) (Fig. 5).

Although a postoperative complication of abdominal wall abscess (Clavien-Dindo classification [11] in grade IIIa) had occurred, it was treated with percutaneous drainage and antibacterial agents. The patient was discharged on postoperative day 29. He refused GC therapy in the postoperative period due to non-haematological adverse events, including general malaise. Alternatively, tegafur/gimeracil/oteracil (S-1) was administered for 1 year after surgery. The treatment regimen consisted of 6-week cycles, in which 80 mg/m^2^ of oral S-1 per day was given for 4 weeks and no chemotherapy was given for the following 2 weeks. There were no adverse events associated with S-1. The patient has been alive and without disease for 6 years after the second surgery.

### Discussion

We report a long-term disease-free survivor after salvage resection with preceding chemotherapy for residual GBC with peritoneal dissemination. Several specific conditions explain this favourable outcome. First, the peritoneal dissemination was very limited. Second, GC therapy yielded an evident tumour shrinkage. Third, and of the most importance, definitive surgery ended in an R0 resection. Fourth, the patient had no other prognostic indicators, including lymph node metastasis, liver metastasis, or bile duct invasion. Lastly, postoperative chemotherapy using S-1 possibly controlled tumour relapse. Overall, aggressive surgery along with systemic chemotherapy benefitted this patient greatly.

The disseminated disease found at referral was induced by the bile contamination associated with the perforation of the gallbladder, which is supported by the fact that disseminated disease was absent during the initial open cholecystectomy. As patients with biliary tract cancers contain floating cancer cells in the bile [12], bile spillage potentially leads to the development of peritoneal dissemination [13]. Several studies [14–16] on this issue demonstrate that early relapse generally occurs after additional resection for the spillage-induced dissemination, leading to dismal prognosis. This observation strongly suggests a treatment strategy that spillage-induced peritoneal dissemination, even if limited, should be treated with systemic chemotherapy, which is consistent with the standardized oncologic approach for distant metastatic GBC. However, the pathophysiological aetiologies between the dissemination derived from the spillage and that from the first presentation may differ. In the former setting, the disseminated disease is often clinically limited due to postoperative adhesions, whereas in the latter setting, dissemination is generally extensive. In addition, the MSTs of 4.8 and 12 months were reported [1, 14], although some bias should be considered.

The first-line chemotherapy using GC for advanced/recurrent biliary tract cancers, including GBC, elicits a 21 to 48% RR with an MST of 4.6 to 11.0 months [17–20]. The second-line S-1 monotherapy elicits a 35% RR with an MST of 9.4 months [21]. Even the sequential use of these two allowed regimens in Japan yields an MST of 8.9–12.5 months in advanced/recurrent disease [22, 23]. If the current patient would have strictly followed the popular strategy using chemotherapy alone, long-term survival could not be expected. Recently, surgical resection has been performed after systemic chemotherapy in patients with initially unresectable biliary tract cancers. Kato et al. [24] reported this challenging surgical approach, termed conversion surgery, where 6 of 14 patients with advanced GBC underwent conversion surgery after gemcitabine-based chemotherapy (gemcitabine monotherapy or GC), which was followed by postoperative chemotherapy, and the R0 status was a key factor to maximize the benefit of this approach. Amemiya et al. [25] reported a 13-year survivor with advanced GBC with periaortic lymph node and hepatic metastases who underwent repeated resections along with systemic chemotherapy. Tomita et al. [9] reported a 3.5-year survivor after GC therapy followed by tumour resection for recurrent peritoneal dissemination in GBC. All these findings suggest that salvage resection with chemotherapy may be of benefit in highly selected patients with advanced/metastatic GBC.

Evident tumour reduction by 3 cycles of GC chemotherapy encouraged us to perform surgery in the present patient. However, as mentioned above, the GC regimen yielded a less satisfactory outcome with a PR rate of 21–48%, and the time to follow up for conversion surgery has yet to be standardized. Further study regarding these problems should be conducted. The surgical procedure employed may be another matter of debate. Based on tumour location, resection of the partial liver, extrahepatic bile duct, and disseminated tumour mass had been considered to be an alternative approach. However, the accurate preoperative staging was challenging in this complex scenario, and we weighed complete clearance of the disease and the removal of the possibly contaminated area from the initial cholecystectomy. Subsequently, we planned an extended resection of the right upper organs including the right liver, extrahepatic bile duct, colon around the hepatic flexure, and duodenum above the ampulla of Vater to enhance the probability of R0 status. Several studies have repeatedly demonstrated that R0 resection is required for prolonged postoperative survival in patients with GBC [25–29]. Therefore, such an extensive surgical approach for complicated disease may be acceptable.

## Conclusions

Although specific clinical factors are closely associated with the favourable outcomes, the present report suggests that aggressive surgery along with chemotherapy may be a promising option in selected patients with highly disseminated GBC, who otherwise have an extremely dismal prognosis.

## Abbreviations

CA19-9: Carbohydrate antigen 19-9
CEA: Carcinoembryonic antigen
GBC: Gallbladder cancer
GC: Gemcitabine plus cisplatin
MDCT: Multidetector-row computed tomography
MST: Median survival time
PET-CT: Positron emission tomography-computed tomography
PR: Partial response
RECIST: Response Evaluation Criteria in Solid Tumours
S-1: Tegafur/gimeracil/oteracil
SUV: Standardized uptake value
UICC: Union for International Cancer Control
